# Systematic review of nephrotoxicity of drugs of abuse, 2005–2016

**DOI:** 10.1186/s12882-017-0794-0

**Published:** 2017-12-29

**Authors:** Kanaan Mansoor, Murad Kheetan, Saba Shahnawaz, Anna P. Shapiro, Eva Patton-Tackett, Larry Dial, Gary Rankin, Prasanna Santhanam, Antonios H. Tzamaloukas, Tibor Nadasdy, Joseph I. Shapiro, Zeid J. Khitan

**Affiliations:** 1 Joan C. Edwards School of Medicine, Marshall University, 1690 Medical Center Drive, Huntington, WV 25701 USA; 2 Aga Khan University Hospital, Stadium Road, Karachi, 74800 Pakistan; 3 The Case Western Reserve University, Cleveland, OH 44106 USA; 4 Johns Hopkins University, Baltimore, MD 21218 USA; 5 University of New Mexico School of Medicine, 87131 Albuquerque, NM USA; 6 The Ohio State University, Columbus, OH 43210 USA

**Keywords:** Nephrotoxicity, Drugs of abuse, Illicit drugs, Acute renal failure

## Abstract

**Background:**

The United States is faced with an unprecedented epidemic of drug abuse. Every year thousands of Americans visit the emergency departments all over the country with illicit drug related complaints. These drugs have been known to be associated with a range of renal pathologies, from reversible acute kidney injuries to debilitating irreversible conditions like renal infarction. So far, no comprehensive study or systematic review has been published that includes the commonly used street drugs and designer drugs with potential nephrotoxic outcomes.

**Methods:**

We conducted a systematic review of published case reports, case series, and cross sectional studies of nephrotoxicities related to drugs of abuse. Literature review was conducted using PubMed/Medline from January 1, 2005 -December 31, 2016 to search for publications related to drug abuse with a defined renal outcome. Publications which reported renal injury in relation to the use of illicit drugs were selected, specifically those cases with raised creatinine levels, clinically symptomatic patients, for instance those with oliguria and proven renal biopsies.

**Results:**

A total of 4798 publications were reviewed during the search process and PRISMA flow chart and Moose protocol regarding systematic reviews were followed. 110 articles were shortlisted for the review. A total of 169 cases from case reports and case series, and 14 case studies were analyzed. Renal manifestations of specific illicit drug abuse were included in this review.

**Conclusion:**

Based on the evidence presented, a wide range of renal manifestations were found to be associated with drug abuse. If the trend of increasing use of illicit drug use continues, it will put a significant percentage of the population at an elevated risk for poor renal outcomes. This study is limited by the nature of the literature reviewed being primarily case reports and case series.

## Background

The United States is stricken by an unparalleled crisis of a drug overdose epidemic. In 2011 the National Center for Health Statistics (NCHS) reported that drug overdose death rates were fivefold higher as compared to the 1980s [1]. A survey by the National Survey on Drug Use and Health (NSDUH) in 2011 reported that 24.6 million Americans of ages 12 or older had used illicit drugs over the past month. This represented approximately 9.4% of the population aged 12 or older [2]. Opioids are the most common cause for deaths related to drug overdose while Marijuana is the most common illicit drug used in the United States [2–4].

According to the Drug Enforcement Agency (DEA), 17.2 billion units of opioids were dispensed at retail level during 2011 compared with 14.9 billion in 2015 [4]. A study estimated that the cost of opioid abuse can go as high as $53.4 billion to cover the legal cost, medical complications, criminal justice system and loss of productivity which is a major portion of the cost and amounts up to $ 42 billion [5]. New psychoactive drugs such as Synthetic Cathinones or Bath Salts are on the rise and patients with their toxicities are presenting to the EDs; they are derivatives of pyrrolidinopropiophenone [MDPV] or mephedrone [6]. They are not commonly tested for during drug screening and are available as various products not yet labeled as controlled substances, allowing a “legal high”. Thus they have a higher affinity for abusers as well as peddlers [4].

There is a wide range of renal manifestations in subjects exposed to elicit drugs ranging from prerenal azotemia to more severe functional and structural injuries. Rhabdomyolysis is a common cause of acute kidney injury (AKI) which is incited by a number of reasons such as prolonged immobilization as seen in opioid users [7]. Rhabdomyolysis is also evident in patients presenting with synthetic cannabinoids and synthetic cathinones abuse but these patients have rhabdomyolysis possibly due to exertional muscular breakdown [8–10].One of the notable causes of renal injury in drug abusers is an increase in the sympathomimetic activity which leads to generalized vasoconstriction and ischemic renal injury; this scenario is usually witnessed in patients with cocaine and methamphetamine abuse [11–13]. Newer opioids and methamphetamines can cause thrombotic microangiopathy (TMA) [14].

Direct renal injury due to illicit drugs have been suggested but most of the reported direct injury is by mode of glomerular immunoglobulin and amyloid deposition such as in heroin abusers [15, 16]. Adulterants such as levamisole which is used to cut cocaine is known for its immunomodulatory properties that can cause antineutrophil cytoplasmic antibodies (ANCA)-vasculitis [17, 18].

To our knowledge, there is no comprehensive report or systematic review that includes the commonly used street and designer drugs with potential nephrotoxic outcomes. In this review, we are summarizing the nephrotoxicities of common and emerging drugs of abuse.

## Methods

### Search strategy

Qualified health care professionals (KM, MK, SS) conducted the literature search in PubMed with the earliest time limit set at January 1, 2005 and latest time limit set at December, 31st 2016. The main literature search was conducted on 11/1/2016 and a secondary update search was carried out on 1/20/2017. Further relevant publications were identified from reference lists and those which fulfilled the inclusion criteria were included. Phrases and MeSH terms used for the search included were acute kidney injury; chronic kidney disease; renal failure; acute renal failure; chronic renal failure; nephropathy. These terms were tested against the names of all drugs under review specifically marijuana, synthetic cannabinoids, Heroin, opioids, vicodin, subutex, suboxone, morphine, methadone, tramadol, opana, norco, oxycontin, hydrocodone, oxycodone, cocaine, cocaine and levamisole, hallucinogens, methamphetamines, 3,4-methylenedioxymethamphetamine (MDMA), foxy, molly, ritalin, adderall, phencyclidine, ketamine, angel dust, Lysergic acid diethylamide (LSD), mescaline, magic mushrooms, peyote mushrooms, bath salts, cathinones, synthetic cathinones, benzodiazepines and Ativan.

### Study inclusion criteria

All abstracts were evaluated by 2 independent reviewers (KM, MK) and the full-text article was procured only if one or both reviewers regarded it as a relevant publication. The reviewers were selected so as to avoid potential conflict of interest due to authorship. Publications which reported renal injury due to illicit drug use were selected, specifically those with 1) raised creatinine levels 2) clinically symptomatic patients for instance those with oliguria 3) proven renal biopsies. All case studies, case reports, case series and original articles (cross sectional, cohorts) in the English language were included. Articles in other languages were only included if a version translated in English was available. Only human based studies with specific renal outcomes attributed to drug abuse were included. CARE guidelines/methodology were adhered to.

## Results

**Fig. 1:**
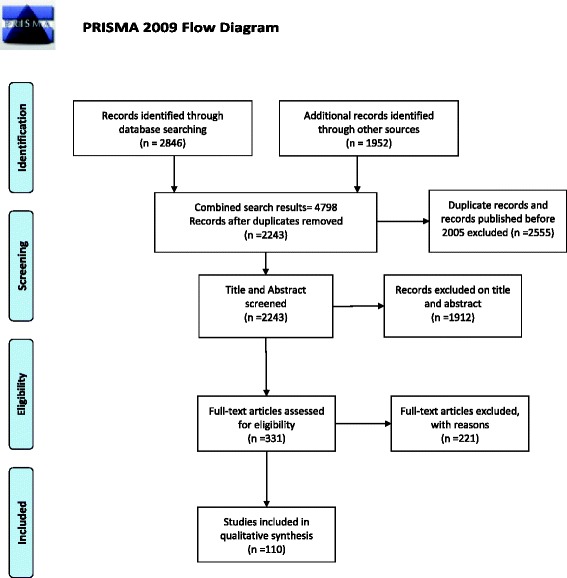
PRISMA 2009 Flow Diagram

A total of 2846 publications were reviewed during the search process through PubMed/Medline while 1952 number of publications were reviewed through the reference list of selected articles. Preferred Reporting Items for Systematic Reviews and Meta-Analyses (Fig. 1 PRISMA) flow chart and Meta-Analysis of Observational Studies in Epidemiology (MOOSE) protocol were followed. 110 articles were shortlisted for the review [19]. Two investigators independently screened all abstracts and assessed the studies for eligibility, then extracted data on specific drugs and their nephrotoxic effects. Selection of articles was based on the title and abstracts but in case of uncertainty, the entire text of an article was read. All literature was catalogued in Mendeley. The primary outcome assessed was the nephrotoxic effects of the abovementioned illicit drugs. A total of 169 cases from case reports and case series with clinical parameters and outcomes reported with or without kidney biopsy were included as shown in Table 1. Table 1 summarizes the different illicit drugs and their patient demographics, showing that a majority of the illicit drug users were male except for Levamisole adulterated cocaine cases. Cases came from a range of ages 2 years to 65 years. Cocaine and Synthetic Cannabinoids (SCB) abuse led to the highest systolic and diastolic blood pressures whereas in other cases there wasn’t such a marked increase or decrease. The pulse was raised with all drugs of abuse and cases mostly presented with gastrointestinal symptoms, hyperthermia, altered mental status or flank pain. Intravenous drug abuse was the most common route used. Where a renal biopsy was performed, the most common outcomes were acute/chronic interstitial nephritis and membranopoliferative glomerulonephritis (MPGN). Amphetamine, Cocaine and levamisole adulterated cocaine users were most likely to need dialysis and amphetamine users were most likely to succumb to death as compared to other illicit drug users.

**Table 1:** Summary of Case reports and Case series of Synthetic Cannabinoids, Bath Salts, Heroin, Amphetamines, Cocaine, Cocaine and Levamisole and CPD – Opioids

	SCB [8, 22, 23, 97–107]	Bath Salts [6, 9, 10, 92, 108–112]	Heroin [15, 16, 35, 37–40, 43, 113]	Amphetamine [13, 81, 85, 86, 88–90, 114–117]	Cocaine [11, 57–63, 66, 71, 118–128]	Cocaine & Levamisole [17, 70, 72–76, 129–133]	CPD Opioids [7, 14, 44, 45, 48, 134–137]
# of Patients In Case Reports	47	11	35	16	22	15	23
Sex (M:F)	45:2 (n = 47)	10:1 (n = 11)	33:2 (n = 35)	12:4 (n = 16)	21:1 (n = 22)	7:8 (n = 15)	2:1 (n = 21)
Age Range (Years)	15–65 (n = 46)	25–45 (n = 10)	24–42 (n = 35)	2–37 (n = 16)	22–65 (n = 22)	22–63 (n = 15)	22–59 (n = 21)
Vitals							
Mean Systolic BP(mm/Hg)	140.9 (n = 21)	135.5 (n = 6)	109.8 (n = 5)	116.1 (n = 8)	170.7 (n = 17)	122 (n = 3)	110.3 (n = 3)
Mean Diastolic BP (mm/Hg)	78.3 (n = 21)	69.3 (n = 6)	68.2 (n = 5)	71.5 (n = 7)	98.2 (n = 17)	73 (n = 3)	59.3 (n = 3)
Mean Pulse (bpm)	91.7 (n = 11)	131.3(n = 8)	99.75 (n = 4)	161.1 (n = 14)	82.5 (n = 11)	96 (n = 3)	124 (n = 3)
Route of Administration							
Smoking	29.8% (n = 14)	0	2.9% (n = 1)	0	59.1% (n = 13)	66.7% (n = 10)	0
Intranasal	0	18.2% (n = 2)	2.9% (n = 1)	0	18.2% (n = 4)	20.0% (n = 3)	0
Oral	14.9% (n = 7)	27.3% (n = 3)	0	87.5% (n = 14)	0	0	26.1% (n = 6)
Intravenous	0	27.3% (n = 3)	88.6% (n = 31)	6.25% (n = 1)	4.5% (n = 1)	6.7% (n = 1)	73.9% (n = 17)
Not Specified	55.3% (n = 26)	27.3% (n = 3)	5.7% (n = 2)	6.25% (n = 1)	18.2% (n = 4)	6.7% (n = 1)	0
Clinical Presentation*							
Gastro-intestinal Symptoms	63.8% (n = 30)	9.1% (n = 1)	2.9% (n = 1)	6.25% (n = 1)	40.9% (n = 9)	26.7% (n = 4)	26.1% (n = 6)
Altered Mental Status	25.5% (n = 12)	36.4% (n = 4)	8.6% (n = 3)	31.25% (n = 5)	4.8% (n = 1)	6.7% (n = 1)	17.3% (n = 4)
Flank Pain	23.4% (n = 11)	0	0	0	27.3% (n = 6)	0	0
Neuro-Muscular Symptoms	6.4% (n = 3)	9.1% (n = 1)	2.9% (n = 1)	0	0	0	13.0% (n = 3)
Hyperthermia	2.1% (n = 1)	36.4% (n = 4)	2.9% (n = 1)	75% (n = 12)	13.6% (n = 3)	6.67% (n = 1)	0
Seizures	2.1% (n = 1)	0	0	56.3% (n = 9)	0	0	0
Other**	2.1% (n = 1)	63.6% (n = 7)	11.4% (n = 4)	12.5% (n = 2)	68.1% (n = 15)	80.0% (n = 12)	21.7% (n = 5)
Lab Parameters							
Mean Peak Serum Cr (mg/dL)	7.6 (n = 43)	7.25 (n = 11)	2.54 (n = 35)	3.31 (n = 16)	7.35 (n = 20)	6.9 (n = 14)	4.21 (n = 23)
Range Peak Serum Cr (mg/dL)	2.6–21 (n = 43)	1.2–15.2 (n = 11)	0.8–11.26 (n = 35)	1.79–9.60 (n = 16)	1.3–17.3 (n = 20)	2–20.8 (n = 14)	1–14 (n = 23)
Range Peak Serum CPK (U/L)	144–301,901 (n = 14)	1183–235,377 (n = 10)	3200–236,000 (n = 3)	863–196,000 (n = 14)	45–990,400 (n = 13)	4585 (n = 1)	17,680–86,000 (n = 4)
# of cases of ADAMTS%^T^ Def.	0	0	0	0	0	0	0
Serology- ANCA	0	0	0	0	0	73.3% (n = 11)	0
# of cases with Urinalysis							
Hematuria	34.4% (n = 11)^+^	80% (n = 4)^B^	61.8% (n = 21)^C^	100% (n = 3)^E^	75.0% (n = 12) ^G^	84.6% (n = 11) ^H^	100% (n = 4)^K^
Proteinuria	68.8% (n = 22)^+^	40% (n = 2) ^B^	94.1% (n = 32) ^C^	100% (n = 3) ^E^	56.25% (n = 9) ^G^	76.9% (n = 10) ^H^	75% (n = 3) ^K^
Eosinophils	12.5% (n = 4)^+^	0	0	0	6.3% (n = 1) ^G^	7.7% (n = 1) ^H^	0
Radiology (U/S, CT Scans)							
Abnormal	45.9% (n = 17) ^A^	66.7% (n = 4)^U^	75% (n = 3)^D^	0% (n = 0)^F^	92.8% (n = 13)^S^	25% (n- = 1)^J^	33.3% (n = 1) ^L^
Kidney Biopsy							
Done	38.3% (n = 18)	0	88.6% (n = 31)	6.25% (n = 1)	54.5% (n = 12)	86.7% (n = 13)	17.4% (n = 4)
Renal Biopsy Diagnosis							
Acute Tubular Necrosis	55.6% (n = 10)	0	0	0	0	0	0
Acute/Chronic Interstitial Nephritis	33.3% (n = 6)	0	9.7% (n = 3)	0	33.3% (n = 4)	7.7% (n = 1)	0
MPGN	0	0	41.9% (n = 13)	0	0	0	0
Thrombotic Microangiopathy	0	0	0	0	25.0% (n = 3)	7.7% (n = 1)	100% (n = 4)
Pauci Immune GN	0	0	0	0	0	76.9% (n = 10)	0
Chronic / Hypertensive	5.6% (n = 1)	0	0	0	16.7% (n = 2)	0	0
Renal Infraction	0	0	0	0	8.3% (n = 1)	0	0
Thrombosis	0	0	0	100% (n = 1)	8.3% (n = 1)	0	0
Crystals	5.6 (n = 1)[22]^M^	0	3.3% (n = 1)[43]^N^	0	0	0	0
Amyloidosis	0	0	32.3% (n = 10)	0	0	0	0
Others	0	0	12.9% (n = 4)^T^	0	8.3% (n = 1)[127]^P^	7.7% (n = 1)[132]^R^	0
Diagnosis of non-biopsy Pts.							
Acute Kidney Injury	29	11	4	14	5	2	19
RPGN^s^	0	0	0	1	0	0	0
Renal Infraction	0	0	0	0	5	0	0
# of Patients Dialyzed	23.4% (n = 11)	27.3% (n = 3)	11.4% (n = 4)	40% (n = 6)	40.9% (n = 9)	40.0% (n = 6)	17.4% (n = 4)
Death as end point	4.25% (n = 2)	9.1% (n = 1)	14.3% (n = 5)	68.8% (n = 11)	0	6.7% (n = 1)	17.4% (n = 4)

* Patients had more than one symptom at presentation;**others include: Dyspnea, SOB, weight loss, chest pain, skin lesions, immobility, urinary complaints like hematuria, anuria; (^+^ 32/47 cases had urinalysis; ^A^ 37/47 cases had imaging studies; ^B^5/11 cases had urinalysis; ^C^ 34/35 cases had urinalysis; ^D^ 4/35 cases had imaging studies; ^E^3/15 cases had urinalysis; ^F^ 2/15 cases had imaging studies; ^G^ 16/22 cases had urinalysis;^H^13/15 cases had urinalysis; ^J^ 4/15 cases had imaging studies; ^K^4/23 cases had urinalysis; ^L^3/23 had imaging studies. ^S^14/22 cases had imaging studies ^U^ 6/11 had imaging studies) ^M^ Calcium oxalate crystals, ^N^ Heroin crystal nephropathy, ^T^ Proliferative GN, Granulomatous GN and MCD + IgA deposits (2,1,1 cases), ^p^ Good Pastures Syndrome, ^R^ Membranous Nephropathy, ^S^ RPGN, ^T^ ADAMTS (A Disintegrin And Metalloproteinase with a Thrombospondin type 1 motif),

Fourteen case studies and analyzed case series were identified as shown in Table 2. A case series based on 456 SCB users showed AKI and rhabdomyolysis as the outcomes when SCB were used solely. Heroin users had a high probability of developing amyloidosis, nephrotic syndrome and progression to end-stage renal disease (ESRD). They also had a higher chance of developing rhabdomyolysis as compared to non- Heroin users and were seen to be co-infected with hepatitis C virus (HCV) as well as Human Immunodeficiency Virus HIV. Controlled prescription drugs (CPD) opioids were significantly shown to lead to renal failure (*p* < 0.001) A post mortem case series on cocaine users showed hypertensive- ischemic nephropathy in most cocaine users. Levamisole adulterated cocaine users were prone to be anti-neutrophil cytoplasm antibodies (ANCA) positive after long term drug use with a 100% prevalence of anti-myeloperoxidase antibodies. Amphetamine case series showed varied effects such as rhabdomyolysis leading to renal failure, malignant hypertension with hypertensive changes on biopsy and in one case series, death of all the subjects due to AKI and rhabdomyolysis.

**Table 2:** List of Clinical studies and Analyzed Case Series of Synthetic Cannabinoids, Bath Salts, Heroin, Amphetamines, Cocaine, Cocaine and Levamisole, CPD Opioids

Author, Year	Type of Study	Results	Misc Findings
Synthetic Cannabinoids			
Reiderer et al., CDC, 2016 [138] n = 456	Cross Sectional	Cases involving Synthetic Cannabinoid Use = 456 Sole agent =277; SCB as agent in multi-agent = 179; AKI in Sole agent SCB = 4.0%; Rhabdomyolysis in Sole agent SCB = 6.1%	N/A
Heroin			
Connolly et al., 2006 [36] n = 20	Cross Sectional	Amyloidosis = 100% Nephrotic Syndrome = 95% ESRD = 60%	Cr mean = 6.4 ± 4.2 mg/dl Proteinuria mean = 7.3 ± 4.1 g/24 h CRP** mean (μmol/l) = 61.4 ± 64
Garg et al., 2011 [41], n = 367	Prospective Cohort Study^+^	Heroin use- HR 1.18 (0.75, 1.87) *p* = 0.43* Model 2- Heroin −1.62 (1.01, 2.60) *p* = 0.045** Model 4- Heroin- 1.28 (1.07, 2.87) *p* = 0.352** Model 5- Heroin- 0.97 (0.53, 3.71) *p* = 0.910**	216 HIV+ HCV Confection cases 151 HCV Monoinfection cases
Kosmadakis et al., 2011 [139] n = 21	Case Control Case =11, Control = 10	Heroin Users (HU) with Rhabdomyolysis = 11 Non-Heroin Users(NHU) with Rhabdomyolysis =10 Rhabdomyolysis Severity HU > NHU	HU v/s NHU CPK^#^ = p 0.039 HU v/s NHU LDH^Z^ = p 0.031 HU v/s NHU PO4 = p 0.002
Novick et al., 2016 [42] n = 2286	Cohort Study	Opiate users = 15%, Cocaine users = 22% Odds of albuminuria: Opiates: OR 1.20, 95% CI 0.83–1.73 Cocaine: OR 1.80, 95% CI 1.29–2.51	Odds of reduced eGFR: Opiates: OR 2.71, 95% CI 1.50–4.89 Cocaine: 1.40, 95% CI 0.87–2.24
CPD Opioids			
Briggs, 2013 [49] n = 33	Case Control Case-15 Control-18	4/8 TTP without infection patients in the case group had renal failure and 7/7 TTP infection patients in the case group had renal failure.	(Odds ratio = 35.0; 95% confidence interval = 3.9–312.1) between TTP-like illness and injection of reformulated Opana ER
Aghabiklooei et al., 2014 [140] n = 322	Cross Sectional	Acute methadone toxicity – Total n = 322, Survivors n-294, Non survivors n- 28 Acute Renal failure: total 16 (4.9%), Survivors 3 (1%), Non Survivors 13 (46.4%) *p* < 0.001	Rhabdomyolysis: Total 15 (4.6%), Survivors 7 (2.4%), Non-Survivors 8 (28.6%)
Glanzmann et al., 2015 [47] n = 200	Case Control Case = 100 Control =100	AKI with morphine – OR- 2.4 (1.02, 6.03) *p* = 0.042 AKI with opioids drug group- OR 3.2 (1.35, 7.75) *p* = 0.008	Age and sex was matched in cases and controls
Cocaine			
Buettner et al. 2014 [55]	Case Series (autopsy)	75/129 cocaine positive subjects Signs of glomerular ischemia with cocaine = OR 3.08 (1.35–7.01) *p* = 0.007 *; OR = 3.34 (1.37–8.14) *p* = 0.01**. Arteriosclerosis with cocaine = OR 2.35 (1.08–5.11) *p* = 0.02*; OR 3.88 (1.49–9.92) *p* = 0.005**	HTN-Ischemic Nephropathy with cocaine = OR 5.42 (1.17–25.20) *p* = 0.02*, OR = 6.0 (1.27–28.44) *p* = 0.02**
Cocaine and Levamisole			
McGrath et al. 2012 [141]	Cross sectional	30/327 New ANCA patients 100% had antimyeloperoxidase antibodies and 50% antiproteinase 3 antibodies, 2 cases had acute renal failure.	N/A
Amphetamines			
Liechti et al., 2005 [142] n = 52	Case Series	90.4% used drugs in combination with Ecstasy 3 patients had rhabdomyolysis and 1 patient had acute renal failure	N/A
CDC 2010 [83]	Case Series	One patient had renal failure, rhabdomyolysis and seizures who was admitted to the ICU and required hemodialysis.	N/A
Jones et al., 2015 [143]	Cross Sectional	Methamphetamine User: N = 47, Malignant Hypertension present in 89.45 (n = 42), CKD was present in 95.7% (N = 45) (55.3% had stage 5 CKD, 8.4% had stage 4 CKD, 10.6% had stage 2 CKD and 12.8% had stage 1 CKD)	Biopsy Findings- Biopsy was performed on 24 patients, Hypertensive changes were present in 50% (N = 12) (with N = 6 had malignant changes), 25% (N = 6) had ESRD, MPGN Type 1 with IgM-C3 deposits was found in 58.3% (N = 14), 37.5% (N = 9) had IgG deposits and 29% (N = 7) had IgA deposits.
Nicol et al. 2015 [87]	Case Series	All subjects’ death. 17/27 died after arrival to hospital. 85% AKI 54% Rhabdomyolysis	Median Peak Cr was 2.4 (1.7–12.8) mg/dl; Median Peak CK was 8200 (1952–237,960) U/L

^+^Several models used to assess risk of AKI due to concern for collinearity between Heroin, cocaine and alcohol

* univariate analysis, ** multivariate analysis

**CRP- C reactive protein, ^Z^ lactated dehydrogenase, ^#^ Creatinine phosphokinase

**Fig. 2:**
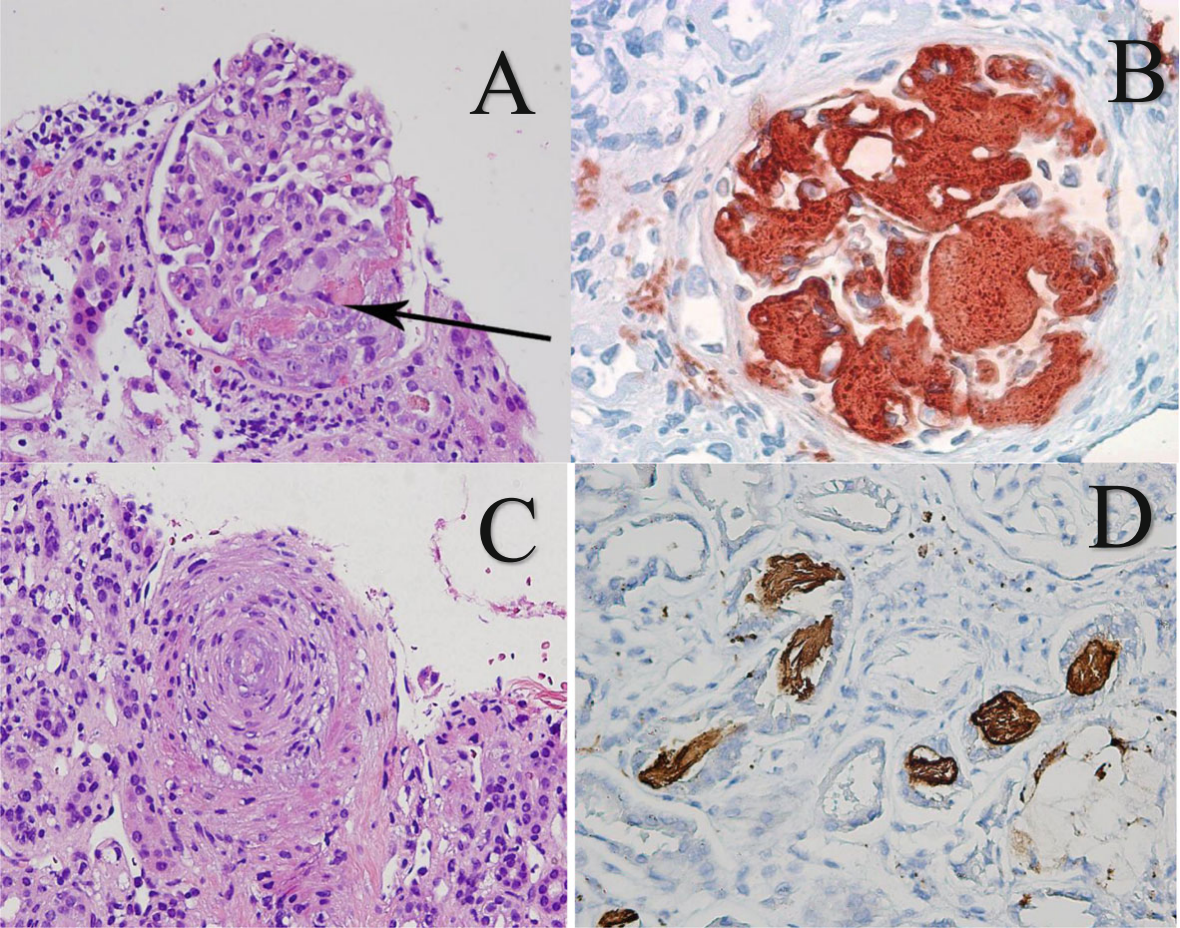
**a** Segmental glomerular necrosis and early crescent formation (arrow) in a heroin abuser who developed glomerulonephritis secondary to MRSA tricuspid endocarditis. There were glomerular IgA and C3 deposits. The patient was also ANCA positive, which may occur in Up to 30% of patients with endocarditis associated glomerulonephritis. **b** Heavy glomerular amyloid A protein deposits (brown color) in an IV drug user and “skin popper” who presented with nephrotic syndrome and was diagnosed with AA amyloidosis. Immunoperoxidase stain with antibody to Amyloid A protein. **c** Severe obliterative vascular changes secondary to chronic stage thromobotic microangiopathy in a young patient with Opana abuse. **d** Myoglobin positive (brown) casts in a young female patient with heavy cocaine use and acute kidney injury. Her CK on presentation was 120,000 and her serum creatinine was 7.9 mg/dl. Immunoperoxidase stain with an antibody to myoglobin

Seven cases of hyperemesis were gathered during the data search with their clinical parameters as shown in Table 3, while 6 cases of *N*-methyl-D-aspartate receptor (NMDA) receptor agonist from two studies were tabulated in as shown in Table 4. Renal biopsy findings in selected patients with drug abuse are shown in Fig. 2. Data presented are specified by type and route (if reported) of drug exposure. Units of laboratory values are all presented in SI units.

**Table 3:** Case reports of Marijuana induced Hyperemesis Syndrome

Author, Year	Age/Sex	Drug	Clinical Presentation	Findings	Findings
Price et al., 2010, [144] n = 1	30/M	Marijuana	Abdominal Pain, Nausea, Vomiting	Cr-3.2 mg/dL Urinalysis-Trace ketones	Acute Kidney Injury
Baron et al., 2011 [145] n = 1	28/M	Marijuana	Vomiting	Cr-5.9 mg/dL	Acute Kidney Injury
Abodunde et al., 2013, [146] n = 1	36/M	Marijuana	Nausea, Vomiting, lethargy	Cr-9.06 mg/dL	Acute Kidney Injury
Chang et al., 2013 [147] n = 1	50/M	Marijuana	Vomiting, epigastralgia, Altered consciousness	Cr-10.1 mg/dL Urinalysis-PH 5.5 with bland sediment	Acute Kidney Injury
Ukaigwe et al., 2014 [148], n = 1	38/M	K-2	Abdominal Pain, Nausea, Vomiting	Cr-4.78 mg/dL,	Pre renal acute kidney Injury
Habboushe et al., 2014 [24] n = 1	25/M	Marijuana	Nausea, Vomiting	Cr-3.21 mg/dL	Acute Kidney Injury
Srihari et al., 2016 [149] n = 1	43/M	Cannabis	Epigastric pain, Nausea, Vomiting	Cr-2.54 mg/dL	Acute Kidney Injury

**Table 4:** Case Report and Case Series of NMDA Receptor Antagonists

Author, year	N, Age/Sex	Drug	Clinical Presentation	Findings	Diagnosis
Wiergowski et al., 2014 [150]	31/M	Methoxetamine, Amphetamines	“lack of Information”	Peak Cr- 3.56 mg/dL Peak CK- 129,800 U/L Anuria +ve	Acute renal failure secondary to rhabdomyolysis
Chenoweth et al., 2015 [96]	27/M	Gacyclidine	Confused, combative	Peak Cr- 1.84 mg/dL Peak CK- 2413 U/L	Acute kidney injury and rhabdomyolysis
	49/M	Gacyclidine	Agitated	Peak Cr- 2.07 mg/dL Peak CK- 28,305 U/L	Acute kidney injury and rhabdomyolysis
	47/M	Gacyclidine	Difficulty in ambulating	Peak Cr- 3.84 mg/dL Peak CK- 13,923 U/L	Acute kidney injury and rhabdomyolysis
	47/M	Gacyclidine	Agitated and confused	Peak Cr- 1.47 mg/dL Peak CK- 1780 U/L	Acute kidney injury and rhabdomyolysis
	47/M	Gacyclidine	Found unconscious next to a gas station	Peak Cr- 5.9 mg/dL Peak CK- 62,694 U/L; Dialysis +ve	Acute kidney injury and rhabdomyolysis

## Discussion

### Marijuana

The psychoactive compound in marijuana is Delta-9-Tetrahydrocannabinol [20, 21]. While synthetic cannabinoids do not have anything in common with marijuana chemically, they can bind to cannabinoid receptors peripherally and in the central nervous system.

Synthetic cannabinoids are known to cause AKI but the exact mechanism is still unclear. As shown in Tables 1&2, several reports have documented specific renal pathologies mainly acute tubular necrosis (ATN) and acute interstitial nephritis (AIN) with some accompanied clinically by rhabdomyolysis. Extreme state of volume depletion because of recurrent vomiting and extreme dehydration along with immobilization can be the precipitating factor.

Bhanushali and Kamel et al. reported that apart from ATN, calcium oxalate crystals were also present upon biopsy. Kamel et al. proposed that as plants are used along with synthetic cannabinoids, there is a possibility that these plants may be oxalogenic and could lead to formation of oxalate crystals which could lead to renal injury. Direct nephrotoxicity of the synthetic cannabinoids remains as yet to be seen [22]. A slightly varied presentation was seen in a patient with Tubulo-Interstitial Nephritis and Uveitis (TINU) syndrome [23].

Although synthetic cannabinoids are the obvious culprits behind these toxicities, chronic use of marijuana can also lead to “Cannabinoid Hyperemesis Syndrome” which results in extreme hypovolemia that leads to severe prerenal azotemia [24] (see Table 3). In general, prerenal AKI in such patients is mostly reversible and remains in question regarding its long term renal sequelae but Coca et al. reported in their systemic review that the chances of developing chronic kidney disease (CKD) increase by 9 fold and the chances of end stage renal disease (ESRD) increase by 3 fold, in comparison to people who have never had an AKI [25].

### Opioids

Heroin use has been known to cause nephropathy since the early 1970s, as reported by the Kings County Hospital in Brooklyn, NY and the term heroin-associated nephropathy (HAN) was coined [26, 27]. Details of reported renal effects of opioids are summarized in Tables 1 & 2.

HAN has been studied in detail but no specific facts have come to light as to whether heroin, morphine, cocaine, adulterants or diseases such as HCV, Hepatitis B virus (HBV) or HIV were responsible for the renal morphological changes [28]. Even though the Heroin epidemic is on the rise, HAN is at an overall decline and it has been debated that HAN predated the investigations of HCV and HIV [29]. It was postulated that chronic use of heroin or its vehicle incites an undefined response which leads to focal glomerulosclerosis with glomerular IgM deposition [30], resulting in nephrotic syndrome [31]. Turgutalp et al. reported a patient who used Heroin thrice a week for 2 years, and on biopsy the patient had minimal change disease with IgA-C3 4+ and IgG 1+ depositions [16].

Do Samerio et al. reported MPGN type I to be the most dominant type of disease present in Caucasian Heroin abusers, but the sample selected for the study had a positive HCV serology [15]. Thus, despite being a valuable resource, their study cannot demarcate whether the nephropathy should be attributed to Heroin use or HCV. Older studies suggest that focal segmental glomerulosclerosis (FSGS) was the most common type of nephropathy in heroin users in the 1970s [27, 32]. Detailed pathologic mechanisms are beyond the scope of this study and can be found elsewhere [29]. Recently, endocarditis associated glomerulonephritis has become an increasingly common disease in IV drug (heroin) users. It is almost always right sided and is associated with variable degree of glomerular immune complex deposition. Crescents can be seen in approximately 50% of the cases and 25 to 30% of patients are ANCA positive. Therefore, differentiating endocarditis-associated crescentric glomerulonephritis from ANCA vasculitis-associated crescentric glomerulonephritis can be difficult, particularly if the glomerular immune complex deposition is mild, which can happen in endocarditis-associated glomerulonephritis. Figure 2a illustrates kidney biopsy of a heroin abuser with segmental glomerular necrosis and early crescent formation.

Amyloidosis has been documented in heroin users [33, 34]. In 2009, a study demonstrated that all 9 patients had renal amyloidosis but all were HCV serology positive [35]. In 2006 Connolly et al. studied renal amyloidosis in intravenous drug users, but their work was limited due to the fact that it was not specified as to whether heroin or cocaine was used, and secondly only patients with amyloidosis were chosen for the study. 90% of the patients had nephrotic syndrome at presentation, 95% patients had HCV antibodies and 5% had HIV [36]. Recently, a case reported by Cooper et al. found serum amyloid A protein deposits and AIN in a Heroin abuser of 18 years [37]. Figure 2b illustrates the kidney biopsy with heavy glomerular amyloid A protein deposits of an IV drug abuser diagnosed with AA amyloidosis.

AKI due to Heroin has been attributed to rhabdomyolysis [38]. A case report by Gupta in 2011 reported rhabdomyolysis associated with AKI [39]. Similarly Abdullah et al. reported rhabdomyolysis and compartment syndrome in their patient who was also concomitantly using cocaine [40]. Grag et al. studied the incidence and predictors of AKI in 367 cases with HCV and HIV coinfection, and reported that after adjustments, Heroin has a slightly significant HR of 1.62(1.01,- 2.60) *p* = 0.05, but loses statistical significance when cocaine use is subsequently added into the model with a hazard ratio (HR) of 1.28 (0.76–2.14)*p* = 0.35 [41]. Meanwhile, a prospective cohort study reported that reduced eGFR was associated with Heroin [42]. Lastly, a relatively new mode of AKI has come to light as “heroin crystal nephropathy” by Bautista et al. in 2015 [43]. This case showed that volume depletion along with elevated urinary pH leads to crystallization of Heroin or its metabolites in renal tubules. Heroin being adulterated with sodium bicarbonate is the likely explanation [43].

It has been reported that Heroin addicts, when put on a methadone rehabilitation program, presented to the emergency department with fatigue and myalgia or at times unconsciousness, were found to have AKI with rhabdomyolysis. Primarily this occurs due to prolonged immobilization. [7, 44, 45].

Another unusual mode of AKI has been reported amongst infants and pediatric age groups; a 27-week-old infant developed hydronephrosis and bladder distension following morphine infusion with normalization of renal function once the infant was catheterized and morphine was stopped [46]. Glazmann et al. reported that the odds ratio (OR) of developing AKI in the pediatric intensive care unit (PICU) when morphine was being administered was 2.4 (1.02–6.03) *p* = 0.04 [47].

In recent years, many cases of oxymorphone or OPANA ER misuse by addicts have been reported. This is achieved by crushing and mixing the pill with water and injecting the said drug. OPANA-ER has been associated with thrombotic microangiopathy (TMA), with or without the presence of infection. A case series by Miller et al. reported 9 out of 18 patients who used oral OPANA-ER intravenously developed a TTP-like condition and had AKI [48]. A study by Center of Disease Control (CDC) in 2013 showed that the odds of developing TMA (TTP-like illness) with misuse of OPANA was OR 35 (3.9–312.1)*p* < 0.01 [49]. Further studies are required to fully understand the roll of OPANA in this condition. Figure 2c illustrates severe obliterative vascular changes secondary to chronic stage TMA in a young patient with Opana abuse.

### Cocaine

Cocaine use has increased in the United States in 2014–2015 due to the increase in cultivation of Coca in Colombia, but these levels are still below the 2006 levels [4]. Cocaine seizures have increased and reached their highest since 2010; this is an indicator for increased cocaine availability. The per gram price of cocaine surged to its highest in the first quarter of 2015, while the purity of cocaine is at its all-time low [4]. Levamisole, dexamisole and fentanyl are the usual adulterants found in cocaine seized by the DEA [4]. Cocaine is available in two forms; as a salt (powder) with HCL and as an insoluble free base which is used as crack [20].

Vasoconstriction caused by cocaine is thought to result from uptake inhibition of catecholamines, serotonin and dopamine and increased release of norepinephrine from the adrenal glands. This surge of catecholamines increases alpha adrenergic stimulation which causes vascular smooth muscles to constrict [50, 51]. Cocaine up regulates vascular endothelin-1 receptors which causes decreased renal blood flow and GFR [29, 52, 53]. Sustained cocaine-induced vasoconstriction has devastating effects that lead to renal hypertension even without signs of systemic hypertension. This hypertensive state causes renal damage and leads to kidney failure [54, 55]. Cocaine may also cause platelet adhesion and microaggregates [56]. Detailed mechanisms and pathogenesis are beyond the scope of this study and can be found in studies by Zimmerman et al. and Jaffe et al. [29, 53]. A combined effect of these proposed mechanisms can cause cocaine-induced renal infarcts (CIRI). Bemanian et al. reported lack of evidence of a thrombus or valvular vegetation and concluded that the vasoconstrictive effect and thrombogenicity of cocaine were the major causes of CIRI [57]. Madhira et al. reported bilateral renal infarction due to vasospasm, confirmed by angiography [58]. Similar cases were also reported [59–62]. TMA has also been reported in relation to cocaine use. Two patients presenting with worsening renal function and hypertension, were shown to have no rhabdomyolysis on investigation, and a kidney biopsy confirmed the diagnosis of TMA [63]. It would be safe to propose that renal infarction should be in the differential diagnosis of severe abdominal pain developing in cocaine users.

Cocaine is associated with rhabdomyolysis [64, 65], but when co-abused with alcohol, liver metabolism leads to the formation of an active metabolite cocaethylene which is highly toxic in comparison to cocaine alone. Recently, a study reported AKI in a patient who co-abused alcohol and cocaine; this report recommended that clinicians should be aware of rhabdomyolysis in patients with a history of co-abuse [66]. Connor et al. reported that rhabdomyolysis was present in 33% of the cocaine users with the prevalence of severe rhabdomyolysis (CK > 10,000 IU/L) being 11% among cocaine users [67]. Renal biopsy findings are that of severe ATN with numerous eosinophilic, globular and frequently pigmented casts, containing myoglobin. Figure 2d illustrates the kidney biopsy finding of a patient suffering from myoglobinuric AKI with a CK of 120,000 U/L and a serum creatinine of 7.9 mg/dL.

Levamisole, a discontinued anti-helminthic drug, is a common adulterant used with cocaine. In 2009, 70% of cocaine in the USA had levamisole. Levamisole when used with cocaine increases the action of catecholamines on neuronal synapses. It also potentiates the reuptake inhibition effect of Cocaine. Adjunctive use of cocaine and levamisole either by smoking or sniffing has been associated with vasculitis [68, 69] Levamisole possesses immunomodulation properties and causes pauci-immune crescentric GN [20]. Usually, this is associated with myeloperoxidase (MPO) and proteinase-3 (PR3) antibodies. In addition, antinuclear antibody (ANA), lupus anticoagulant and low complement levels are detected in most patients [70–76]. Immune complex glomerulonephritis (GN) has been described but in such case, an underlying infection should always be considered [20, 74]. A case by Neynaber reported that their patient developed a fulminant case of granulomatosis with polyangitis and PR3 antibody [17].Though the synergism between these two has not yet been fully understood, the toxicity of levamisole and cocaine combined, expedites their respective manifestations leading to severe disease. In Tables 1 & 2, we have listed case reports and studies where the renal effects of cocaine in different scenarios have been studied.

### Methamphetamine

Methamphetamines especially MDMA are known to cause AKI by several mechanisms. The most common mechanism, also found to be the most prevalent throughout literature selected for this study, was myoglobinuria-associated tubular injury secondary to rhabdomyolysis [77]. Other commonly proposed renal effects include prerenal azotemia, malignant hypertensive nephropathy, hyponatremia and necrotizing vasculitis, while some novel mechanisms include TTP induced by MDMA and thrombosis [13, 78–84].

Ago et al. reported a patient who collapsed minutes after he was injected with methamphetamine. Multi-organ failure and rhabdomyolysis followed, and a biopsy showed presence of myoglobin pigmentation in his kidneys and AKI [85]. Similarly, Lin et al. reported a patient who ingested 26 tablets of Ecstasy resulting in a tonic-clonic seizure and presented with hyperthermia [86]. CPK is a good indicator for the extent of rhabdomyolysis, which in our literature review ranged from 1672 IU/L to 196,000 IU/L in patients presenting with AKI. A case series documented that 18 of 27 patients had AKI and 50% of them had rhabdomyolysis [87]. Santoro et al. reported a dextroamphetamine user, whose intense workout routine led to the development of rhabdomyolysis but timely treatment protected her from renal injury [82]. Another cause of rhabdomyolysis reported by Davis et al. was attributed to serotonin syndrome [88].

Thrombosis, TTP and coagulopathy have also been reported in association with MDMA. Eldehni et al. reported a 22 year old male with worsening renal function and bilateral loin pain who was found to have a small venous thrombus in the corticomedullary junction [85]. Though the exact mechanism is not known, it would be fair to postulate that MDMA was the exacerbating agent in this case as it is known to cause coagulopathy combined with its sympathomimetic effects. Disseminated intravascular coagulopathy (DIC) has been reported as a common occurrence along with renal failure; five out of six AKI patients in a case series had DIC as well [89]. Recently reported, a patient after consuming Ecstasy rapidly developed AKI, rapidly progressive glomerulonephritis (RPGN) and TTP with a platelet count as low as 5000 /uL, followed by the patient’s death [90]. De Fallois postulated that there is similarity in the structure of MDMA and thienopyridines which are known to cause TTP. Necrotizing vasculitis from MDMA leading to ESRD was also reported in one patient by Bingham [84].

### Bath salts – Synthetic Cathinones

AKI with mild rhabdomyolysis and hyperuricemia secondary to ingestion of bath salts has been reported and urinary sediment analysis revealed ATN [6]. Regunath et al. reported a patient who developed oliguric AKI coupled with mild rhabdomyolysis after ingesting bath salts. In this case supportive measures weren’t enough and continuous renal replacement therapy was required for a period of 48 h [9]. There have been more severe cases that resulted in DIC and rhabdomyolysis [91].

Multi organ failure has also been known to occur after injection of bath salts, with anuric AKI being the primary presentation. Continuous renal replacement therapy was required, followed up with intermittent therapy for a span of one month [92].

### NMDA receptor antagonists

NMDA receptor antagonists, potent hallucinogens, are primarily used for their euphoric, anesthetic and hallucinogenic properties. PCP and Methoxetamine mostly cause AKI secondary to rhabdomyolysis, while Ketamine has been known to cause lower urinary tract dysfunction [77]. Peng et al. reported a 45-year-old male intranasal Ketamine user who had increased urinary frequency with urethral pain. In this patient, computed tomography showed bilateral hydronephrosis and decreased bladder capacity confirming the diagnosis of Ketamine induced uropathy [93]. Similar cases were also reported [94, 95].

As shown in Table 4, several reports have documented renal injury as a result of NMDA receptor antagonists. A case series by Cheoweth et al. based on Gacyclidine users (which is similar to Phencyclidine), reported that all patients had rhabdomyolysis and AKI [96].

## Conclusion

The use of illicit drugs continues to surge and is approaching epidemic proportions throughout the United States. With nearly 10% of the population older than 12 having taken drugs for nonprescription purposes in the last month (2), new synthetic substances being introduced into the market and increased ease of access there will undoubtedly be a continual increase in the negative medical, financial and societal outcomes. The pathophysiology underlying renal injury from drugs of abuse continues to be delineated as research into this condition develops. Studies performed to date have elucidated specific mechanisms of injury. However, many of the studies are confounded by multiple illicit substance or comorbid conditions such as HIV or hepatitis. Continued resources, education and research are needed to fully understand the myriad of renal insults related to drugs of abuse.

## Abbreviations

CPD: Controlled prescription drugs
MDMA: 3,4-methylenedioxymethamphetamine
MDPV: Pyrrolidinopropiophenone
NCHS: National Center for Health Statistics
NSDUH: National Survey on Drug Use and Health
TTP: Thrombotic thrombocytopenic purpura
NMDA: *N*-methyl-D-aspartate receptor
AKI: Acute kidney injury
CPD: Controlled prescription drugs
ATN: Acute tubular necrosis
AIN: Acute interstitial nephritis
TINU: Tubulo-interstitial nephritis and uveitis
CKD: Chronic kidney disease
ESRD: End stage renal disease
HAN: Heroin-associated nephropathy
HCV: Hepatitis C virus
HBV: Hepatitis B virus
HIV: Human immune deficiency virus
MPGN: Membranoproliferative glomerulonephritis
FSGS: Focal and segmental glomerulosclerosis
TMA: Thrombotic microangiopathy
CIRI: Cocaine-induced renal infarcts
MPO: Myeloperoxidase
PR-3: Proteinase-3
ANA: Antinuclear antibody
GN: Glomerulonephritis
RPGN: Rapidly progressive glomerulonephritis
DIC: Disseminated intravascular coagulopathy
HR: Hazard Ratio
CDC: Center of Disease Control
PICU: pediatric intensive care unit
DEA: Drug Enforcement Agency (DEA)
OPANA ER: Extended release
PRISMA: Preferred Reporting Items for Systematic Reviews and Meta-Analyses
MOOSE: Meta-Analysis of Observational Studies in Epidemiology
SCB: Synthetic Cannabinoids
ANCA: Anti-Neutrophil Cytoplasm Antibodies
TINU: Tubulo-interstitial nephritis and uveitis syndrome
ANA: Antinuclear antibodies
